# An explorative study of school performance and antipsychotic medication

**DOI:** 10.1186/s12888-016-1041-0

**Published:** 2016-09-21

**Authors:** J. van der Schans, S. Vardar, R. Çiçek, H. J. Bos, P. J. Hoekstra, T. W. de Vries, E. Hak

**Affiliations:** 1 Department of Pharmacy, PharmacoTherapy, −Epidemiology & -Economics, University of Groningen, Antionius Deuginslaan 1, Groningen, 9713 AV The Netherlands; 2 Department of Psychiatry, University of Groningen, University Medical Center Groningen, Hanzeplein 1, Groningen, 9713 GZ The Netherlands; 3 Department of Pediatrics, Medical Center Leeuwarden, Henri Dunantweg 2, Leeuwarden, 8934 AD The Netherlands; 4 Department of Epidemiology, University Medical Center Groningen, Hanzeplein 1, Groningen, 9713 GZ The Netherlands

**Keywords:** Antipsychotic treatment, Academic performance, Children, Pharmacoepidemiology

## Abstract

**Background:**

Antipsychotic therapy can reduce severe symptoms of psychiatric disorders, however, data on school performance among children on such treatment are lacking. The objective was to explore school performance among children using antipsychotic drugs at the end of primary education.

**Methods:**

A cross-sectional study was conducted using the University Groningen pharmacy database linked to academic achievement scores at the end of primary school (Dutch Cito-test) obtained from Statistics Netherlands. Mean Cito-test scores and standard deviations were obtained for children on antipsychotic therapy and reference children, and statistically compared using analyses of covariance. In addition, differences in subgroups as boys versus girls, ethnicity, household income, and late starters (start date within 12 months of the Cito-test) versus early starters (start date > 12 months before the Cito-test) were tested.

**Results:**

In all, data from 7994 children could be linked to Cito-test scores. At the time of the Cito-test, 45 (0.6 %) were on treatment with antipsychotics. Children using antipsychotics scored on average 3.6 points lower than the reference peer group (534.5 ± 9.5). Scores were different across gender and levels of household income (*p < 0.05*). Scores of early starters were significantly higher than starters within 12 months (533.7 ± 1.7 vs. 524.1 ± 2.6).

**Conclusion:**

This first exploration showed that children on antipsychotic treatment have lower school performance compared to the reference peer group at the end of primary school. This was most noticeable for girls, but early starters were less affected than later starters. Due to the observational cross-sectional nature of this study, no causality can be inferred, but the results indicate that school performance should be closely monitored and causes of underperformance despite treatment warrants more research.

## Background

Childhood psychiatric disorders are of concern because of the associated negative impact on general wellbeing across the lifespan, for example, lower educational achievements [1–5]. The findings in several studies suggest that the adverse impact of childhood onset psychiatric disorders on educational attainment is largely accounted by problems of inattention and conduct. Most psychiatric disorders present symptom patterns that cause severe impairment on the emotional, cognitive and social level, resulting into the child being unable to carry out his/her educational potential. Esch et al. [4] and Kumpulainen et al. [2] studied the relationship between psychiatric symptoms and performance level at school among 8-year-old children. Children facing psychiatric difficulties were more likely to receive extra tutoring or special education. The probability of getting special education was highest for attention deficit disorders, anxiety, and oppositional/conduct disorders. Children from low socio-economic status families had more psychiatric symptoms and performed less successfully at school than children from more advantaged environments. This suggests that children with psychiatric symptoms or disorders are more likely to have lower performance levels at primary school.

A few studies have shown that psychiatric disorders in children and adolescents can negatively impact academic performance. Medical drug treatment with antipsychotics has been proven effective in reducing symptoms of such disorders.[6] However, it remains uncertain whether such treatment restores academic performance since a systematic comparison between drug treated children and their peers is currently lacking. Insights into academic performance among this vulnerable group of children is essential to further optimize care, their school performance and general well-being. We therefore compared school performance among children using antipsychotic drugs at the end of primary education with their peers using an observational cross-sectional design. We also explored differences across various relevant subgroups according to sex, ethnicity, parent household, and household income as well as early versus late start of drug treatment.

## Methods

### Study setting

This retrospective cohort study was performed by linking drug prescription data from the University Groningen pharmacy prescription database ‘InterAction Database’ (IADB.nl) to data obtained from Statistics Netherlands. The IADB database is a longitudinal pharmacy-dispensing database with detailed patient-based drug prescription data from 1994 till 2012 from approximately 600,000 patients in the northern Netherlands. Prescription rates amongst the database population have been found to be representative of the Netherlands as a whole, and the database has been widely used for research [7]. Each prescription record contains information on the date of dispensing, the quantity dispensed, the dose regimen, the number of days the prescription is valid, the prescribing physician and the Anatomical Therapeutic Chemical code (ATC code). Each patient has a unique anonymous identifier and therefore informed consent was not necessary; date of birth and sex are known. Due to high patient pharmacy commitment in the Netherlands, the medication records for each patient are virtually complete [8]. Access to the database for this specific study was granted by the supervisory board of the IADB.

Statistics Netherlands is a Third Trusted Party for data linkage in The Netherlands. Datasets such as the Cito-test, an academic achievement score-test, database and the IADB.nl database are assigned an unique personalized identification code based on identification information such as birth date or social security number. Statistics Netherlands performed the linkage between datasets and removed all identification information from the dataset which could be accessed by the researchers, hence researchers are unable to identify patients after linkage of the data. We obtained datasets covering ethnicity, type households, and income of households. Due to national regulations confidentiality was warranted by following the guidelines of disclosure of identities of individual persons, enterprises, institutions or households by Statistics Netherlands. Access to the Statistics Netherlands data and linkage with the IADB was given by the Customised Services Team of Statistics Netherlands.

Our study population consisted of 7,994 children born in 1996–2001 from the IADB database, whose outcome Cito-test scores were available in the Statistics Netherlands database. For the cross-sectional design at the end of primary education, we obtained drug prescription data from January 1^st^ 2001 through March 31^st^ 2012.

Individual Cito-test scores from the years 2009–2012 and demographic data on every child, including sex, ethnicity, parent household, and household income were obtained from Statistics Netherlands. We used data registered until 2012, which represented the most recent years of the IADB prescription data available at the time of our study. Household income data of the year 2011 was used, which represented the mid-term of the period in which the Cito-test scores were available. Also, we obtained parent household information of the year the Cito-test. We categorized ethnicity as Dutch or non-Dutch, in which the classification was based on the mother’s country of birth. Parent household was classified as being a one-parent or a two-parent household. The standardized disposable household income in percentiles from Statistics Netherlands was used to classify household income and defined as the gross income of all household members minus paid income transfers, social contributions and taxes. The disposable household income was adjusted for the individual family members to facilitate comparison between the various types of households. The standardized disposable household income was subsequently apportioned to each of the household members. In this article household income was defined as the standardized disposable household income. We classified household income in three groups: low, middle and high; the maximum year income of the low income is € 17 374 and the maximum year income of the middle group is € 25 212.

### Definitions of children on antipsychotic treatment and reference group

#### Children on antipsychotic drug treatment

**Fig. 1:**
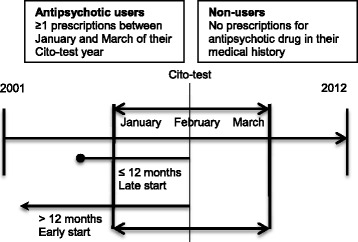
Study design of the different comparator groups

We assumed children to have been treated if they had a prescription for any antipsychotic drug in the year the Cito-test was taken from January 1^st^ to March 31^st^ (Fig. 1). We refer to this treatment group as antipsychotic users. For each treated child, all prescriptions for antipsychotic drugs *(ATC: N05A)* were identified from the IADB database. The date of first dispensing of any antipsychotic drug marked the start of treatment. We also included children with a prescription in December the year before the Cito-test was taken, who also had a prescription in April the year the Cito-test was taken. These children had a prescription for at least 100 days and therefore probably used antipsychotics during the period of the test. Children were only included in the study when they were present in the database for at least 1 year before initiation of antipsychotic treatment.

#### Start of treatment

The initiation of antipsychotic treatment for each child was defined by the date of the first dispensing of a prescription for any antipsychotic drug within the study period (2001–2012). We compared school performance within the antipsychotic users group according to their different timing of initiating drug treatment before the Cito-test, i.e. children with early vs. late start of treatment. We used February in the year the Cito-test as reference date and we defined antipsychotic users as starting the treatment within 12 months (late start) and earlier than 12 months (early start) (Fig. 1).

#### Reference peer group

We selected children from the IADB database born between 1996 and 2001 with no prescriptions for any antipsychotic drug in their medical history. They were referred to as non-users.

### Academic school performance

Academic performance was operationalized into Cito-test scores available from Statistics Netherlands. The Cito-test, is a nationwide used independent, standardized, academic achievement score-test which informs about the most suitable type of secondary education (http://www.iaea.info/documents/paper_1162d212f6.pdf). The score is an indicator for the learning achievement of a child; indirectly the score is an indicator of his or her intelligence, motivation, concentration, and drives to learn (http://www.iaea.info/documents/paper_1162d212f6.pdf). The test is taken in around 85 % of the Dutch primary schools in the first week of February each year. Standardized Cito-test scores range from 501 to 550, based on a transformation of the number of correct answers for two-hundred multiple-choice questions covering language, arithmetic/mathematic, and study skills, which indicates the most suitable type of secondary education (≤523: secondary education leading to vocational education level 3, 524–528: level 2, 529–536: level 1, 537–544: secondary education leading to higher professional education, ≥545: secondary education leading to university) (http://www.iaea.info/documents/paper_1162d212f6.pdf). The language part of the Cito-test consists of spelling, reading comprehension, verbs, vocabulary, and writing. Arithmetic/mathematic questions cover measurements, time, money, fractions, percentages, and ratios. Study skills questions involve the child’s ability to process information from dictionaries, tables, graphs, scheme’s, maps etc.

### Statistical analysis

We first conducted descriptive explorative analyses to assess the frequencies of covariates within our antipsychotic and non-users population. Pearson chi-square tests were used to compare the frequencies of the baseline characteristics between comparison groups. In subgroup analyses, we described crude mean Cito-test scores with standard deviations for the subgroups and tested for between-group difference with analysis of variance (ANOVA) (two-sided). Then we performed multivariate analysis to describe differences school performance related to children with or without antipsychotic treatment across the levels of the subgroups. We applied analysis of covariance (ANCOVA) models to estimate mean Cito-test scores across the different groups (users vs. non-users; early vs. late starters), initially unadjusted and also subsequently adjusted for sex, ethnicity, parent household and household income. A *p* value <0.05 was considered statistically significant. All analyses were performed using SPSS 20.0.

## Results

**Fig. 2:**
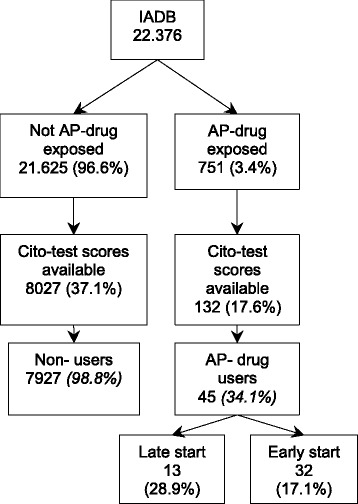
Flowchart of the study population. Abbreviation: AP, anti-psychotic

Out of the 7994 children registered in the IADB.nl who could be linked to their Cito-test scores, 45 (0.6 %) children were treated with an antipsychotic drug at the time of the Cito-test of whom 36 were boys (antipsychotic users, see Fig. 2). Among all children in the total IADB population, the availability of Cito-test scores was lower for the antipsychotic drug treated population (17.6 %) than for the reference population (37.1 %). In addition, Fig. 2 shows that 32 (71 %) children were early starters defined as starting treatment with antipsychotics earlier than 12 months ago, and 13 (17.6 %) were late starters.

Table 1 shows that 27 (60.0 %) of the antipsychotic users had prescriptions for risperidone, followed by 8 (17.8 %) for pipamperone, 7 (15.6 %) for aripiprazole, 2 (4.4 %) for both risperidone and pipamperone, and 1 (2.2 %) for quetiapine. Baseline characteristics among our study population varied only for the subgroup sex. Boys comprised 47.3 % of non-users and 80.0 % of antipsychotic users. Overall, boys were more frequently treated with antipsychotic drugs than girls. No difference in the distribution of the frequencies within levels of the subgroups between antipsychotic users and reference group was found for the subgroups ethnicity, parent household and household income.

**Table 1:** Comparison of the baseline characteristics between non-users and antipsychotic users

	Non-users, n (%)	Antipsychotic users, n (%)	*p* value (Chi2)
Total	7927 (100)	45 (100)	
Prescriptions:			
Risperidone		27 (60.0)	
Pipamperone		8 (17.8)	
Aripiprazole		7 (15.6)	
Risperidone + Pipamperone		2 (4.4)	
Quetiapine		1 (2.2)	
Start of treatment:			
Late <12 months		13 (28.9)	
Early > 12 months		32 (71.1)	
Subgroups:			
Sex			<.05*
Boys	3748 (47.3)	36 (80.0)	
Girls	4179 (52.7)	9 (20.0)	
Ethnicity			.695
Dutch	7018 (88.5)	39 (86.7)	
Non-Dutch	909 (11.5)	6 (13.3)	
Parent household			.141
Two-parent household	6486 (81.8)	33 (73.3)	
One-parent household	1441 (18.2)	12 (26.7)	
Household income			.134
Low	2674 (33.7)	20 (44.4)	
Middle	3038 (38.3)	18 (40.0)	
High	2215 (27.9)	7 (15.6)	

*Abbreviation*: *n*, sample size

* Significant at .05 level

In univariate analyses children using antipsychotics at the time of the Cito-test scored on average 3.6 points lower (*p* < .05) on their Cito-test compared with the non-using general population (Table 2). No significant difference was detected between the mean Cito-test scores of boys in the users group and boys in the non-users group. However, girls in users group had significantly lower average Cito-test scores (525.2 ± 7.5) than girls in reference group (534.1 ± 9.5). The subgroup of Dutch children using antipsychotic drugs during the Cito-test (531.6 ± 10.4) performed worse than Dutch children who did not use antipsychotic drugs (534.9 ± 9.4). The difference in mean Cito-test scores between non-Dutch users and non-Dutch non-users was not statistically significant. Also, both groups did not differ with respect to parent household and low household income. There was a significant difference between mean Cito-test scores of users and non-users in middle (non-users: 534.3 ± 9.2 vs. antipsychotic users: 529.7 ± 10.8) and high household income (non-users: 538.1 ± 8.4 vs. antipsychotic users: 530.9 ± 9.2). Antipsychotic users who started their treatment late (≤12 months) had a lower average score (524.9 ± 9.1) than those who started early (533.4 ± 10.1).

**Table 2:** Results of univariate and multivariate analyses in relation to Cito-test scores including the subgroups

	Non-users M ± SD	Antipsychotic users M ± SD	Subgroups *p* value	Interaction *p* value
Total	534.5 ± 9.5	530.9 ± 10.5	< .05*	
Subgroups:				
Sex				.075
Boys	535.0 ± 9.4	532.4 ± 10.7	.100	
Girls	534.1 ± 9.5	525.2 ± 7.5	< .05*	
Ethnicity				.785
Dutch	534.9 ± 9.4	531.6 ± 10.4	< .05*	
Non-Dutch	531.9 ± 10.0	526.8 ± 11.2	.219	
Parent household				.301
Two-parent household	535.0 ± 9.4	532.1 ± 10.6	.075	
One-parent household	532.4 ± 9.5	527.8 ± 9.9	.099	
Household income				.037*
Low	531.9 ± 9.7	532.1 ± 11.0	.940	
Middle	534.3 ± 9.2	529.7 ± 10.8	< .05*	
High	538.1 ± 8.4	530.9 ± 9.2	< .05*	
Start of antipsychotic treatment:				
Late start (≤12 months)		524.9 ± 9.1	<. 05*^a^	
Early start (>12 months)		533.4 ± 10.1		

*Abbreviations*: *n* sample size, *M* mean, *SD* standard deviation

* Significant at .05 level

^a^ Comparison within the antipsychotic user group

In multivariate analysis, Cito-test scores of non-users and users were independently and statistically different across levels of household income (*p* value .037). Although, children from one-parent households performed lower than two-parent households, this finding was not statistically significant. The same applied for children with non-Dutch ethnicity vs. Dutch ethnicity.

After adjustments for the measured baseline differences between the comparison groups, the means of Cito-test scores remained significantly different between the antipsychotic users (531.4 ± 1.4) and reference group (534.5 ± .1), *p* value .024 (Table 3). The adjusted mean estimates remained similar to the crude mean Cito-test scores and indicated little differences across the levels of the subgroups. However, the difference between late starters and early starters was statistically significant. Compared with early starters (533.7 ± 1.7) we found a significantly lower mean Cito-test score for the late starters (524.1 ± 2.6).

**Table 3:** Crude and adjusted mean Cito-test scores adjusted for baseline differences of sex, ethnicity, parent household and household income with ANCOVA analyses

	School performance			
	Crude M ± SD	Adjusted M ± s.e.	n	*p* value
AP treatment				
Antipsychotic users	530.9 ± 10.5	531.4 ± 1.4	45	.024*
Non-users	534.5 ± 9.5	534.5 ± .1	7927	
Treatment with:				
MPH	531.9 ± 9.5	531.9 ± .6	272	.882
MPH + AP	532.2 ± 10.2	532.2 ± 2.0	22	
Start of AP treatment				
Late start ≤ 12 months	524.9 ± 9.1	524.1 ± 2.6	13	.004*
Early start > 12 months	533.4 ± 10.1	533.7 ± 1.7	32	

*Abbreviations*: *AP* antipsychotic drug, *M* mean, *SD* standard deviation, *s.e.* standard error, *n* sample size

*: Significant at the .05 level

## Discussion

The main finding of this explorative study is that children using antipsychotics at the time of the Cito-test show lower academic achievement at the end of primary school than their peer group. Importantly, girls seem to be more affected than boys, but those who started earlier showed similar performance as their peers. To our knowledge, these data are the first to describe the primary school performance among children on antipsychotic therapy.

Our results are largely in agreement with earlier findings reported by Kumpulainen et al. [2] who demonstrated in a questionnaire study that children having psychiatric symptoms are more likely to be low achievers in school, though it was unknown whether the study subjects were on drug treatment.

A strength of this study is that we used a nationwide used, validated and standardized test to determine the school performance of children and did not rely on self-reported questionnaires. Information bias is highly unlikely. Each study year, the Cito-test was taken at the same time by the similar amount of children with a similar average score and the test results are comparable over the years (http://www.cito.nl/onderzoek%20en%20wetenschap/achtergrondinformatie/primair_speciaal_onderwijs/eindtoets_onderzoek_achtergrond). It is an objective, quantitative, standardized achievement test, which represents the learning achievement of a child. Because we used records from Statistics Netherlands our data is not influenced by recall bias or subjective measures often associated with data reported by parents or children. Furthermore, we used pharmacy-dispensing records which is a valid method to obtain information on actual prescription drug use. Also, it was possible to follow children from the moment of first dispensing to the year in which the Cito-test was taken. The availability of prescription history information for at least 1 year before the Cito-test allowed the possibility for differential exposure, i.e. early starters versus late starters and concurrent treatment.

An important feature of the cross-sectional nature of this study design is that we cannot draw causal conclusions from these data. Several studies reported a negative association between psychiatric disorders and educational attainment and school dropout, and the present findings may indicate that there is already a notable difference present at the end of primary school [1, 3–5]. Children using antipsychotics may face learning difficulties because of their underlying psychiatric disorder which may prevent them from carrying out their educational potential. We did not have information on the severity of symptoms, underlying diagnoses, or indications, for which the drugs were prescribed. Importantly, our study lacks information about possible concurrent psychosocial interventions or educational school services received by children in the study population. While it is possible that their underlying illness is related to a lower academic performance, it remains uncertain whether antipsychotic treatment can be a mediating or causing factor.

A further limitation of our study was the number of children receiving antipsychotic treatment being relatively small due to low prevalence of psychiatric disorders and relatively low number of children that could be linked to the Cito-test scores. Therefore, smaller differences in scores could in some subgroups not be adequately tested.

The prevalence rate of antipsychotic use found in our study (6 per 1000) is in accordance with the reported prevalence of 6,8 per 1000 in 2005 by Kalverdijk et al. [9]. In comparable studies from European countries researchers reported overall prevalence rates of 3.2 per 1000 in Germany in 2012, 3.4 per 1000 in France in 2004 and 10,6 per 1000 in Iceland in 2007 [10–12]. Hence, we might conclude that our population is representative for the Netherlands as a whole. Importantly, over the years 2009–2012, children in group eight of all primary schools who participated in the Cito-test in the Netherlands had an average overall Cito-test score of 535, which is similar to the average of the reference group in our study population. In addition, the age and gender distribution of the general population corresponds with the population of the IADB, which indicates that both populations are similar.

A notable difference was the availability of Cito-test scores between the drug treated population (17.6 %) and the reference population (37.1 %). The low number of Cito-test data can be attributed to the fact that before 2010 the test data reported to Statistics Netherlands was incomplete due to the availability of the data by a secondary party. This difference between the antipsychotic group and the reference group can be explained by the fact that some children are not obliged to take the Cito-test in case of severe learning difficulties. For this reason, the difference in availability of Cito-test scores will probably give an underestimation of our results.

Antipsychotic treatment was mainly observed in boys and to a lesser extent in girls which is consistent with several studies that have reported that antipsychotic treatment of children predominantly involves male patients and mainly involves the treatment of disruptive behavior disorders [9, 12, 13]. Disruptive behavior disorders are more commonly diagnosed in boys than in girls [14]. Importantly, the differences in school performance between anti-psychotic users and reference group was more pronounced among girls than among boys. Girls seem to be primarily responsible for the significantly poorer school performance detected for antipsychotics users. Although no causal conclusions can be drawn from this study, it seems unlikely that the beneficial effect of antipsychotic drugs is different for pre-puberal boys than girls. Our data suggest that the indications for antipsychotic treatment may be different across sex and therefore girls may respond differently to their treatment. Consequently, antipsychotic drugs may be less effective for those specific indications. Such sex differences in indications for antipsychotics and school performance outcomes need to be further explored in future studies.

There is a lack of knowledge about the most preferable initiation and duration of antipsychotic treatment in childhood. In our data children starting antipsychotic treatment earlier than 12 months before the testing period indicated a better average school performance outcome than late starters. This suggests that children starting treatment earlier are more likely to have had more opportunity to adjust and to be treated with the optimal antipsychotic drug. However, for the interpretation of these results we lack information about the development of their psychiatric disorders at different ages and different symptom patterns that may influence their school performance.

The results of this study suggest that children with antipsychotic treatment perform lower on primary school and this special group of children may be unable to carry out their educational potential. Antipsychotic treatment does not appear to entirely restore academic performance associated with psychiatric disorders. This highlights the importance of additional (non-pharmacological) interventions aimed at academic functioning, notably among girls. This information regarding the school performance in this specific population could be useful for parents, teachers, and clinicians. Future research needs to focus on possible reasons for their susceptibility to underachievement on primary school. Furthermore, the long-term effects of antipsychotic use in children should be examined.

## Conclusion

In conclusion, this first exploration showed that children on antipsychotic treatment have lower school performance compared to the reference peer group at the end of primary school. This was most noticeable for girls, but early starters were less affected than later starters. Due to the observational cross-sectional nature of this study, no causality can be inferred, but the results indicate that school performance should be closely monitored and causes of underperformance despite treatment warrants more research.

## Abbreviations

ADHD: Attention-Deficit/Hyperactivity Disorder
ATC code: Anatomical Therapeutic Chemical code
IADB: Interaction database
